# Supplementary material for: Gekko gecko as a model organism for understanding aspects of laryngeal vocal evolution
Source: J Exp Biol. 2024 Jul 31;227(15):jeb247452. doi: 10.1242/jeb.247452 (PMC11418165; doi:10.1242/jeb.247452)
Supplement: Supplementary information [file jexbio-227-247452-s1.pdf]

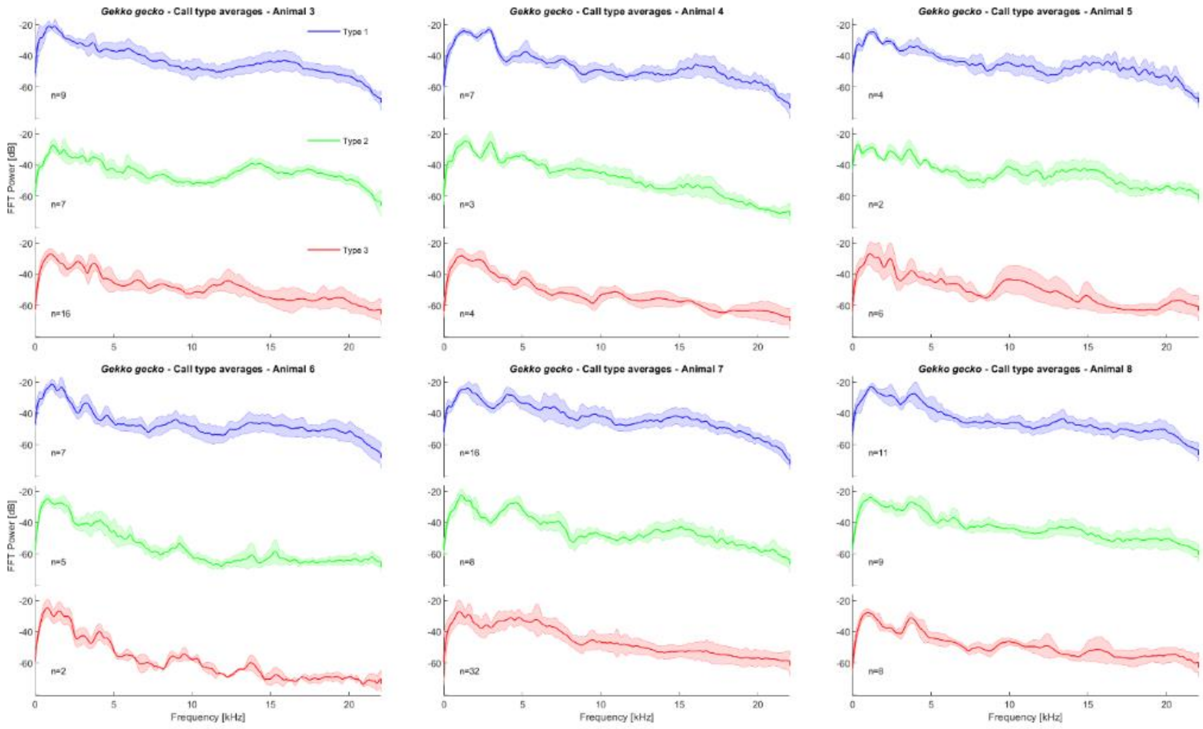

Fig. S1. Mean frequency spectra of the three call types produced by each individual animal. Solid lines show averages and shaded areas depict ± 1 standard deviation.

Journal of Experimental Biology • Supplementary information

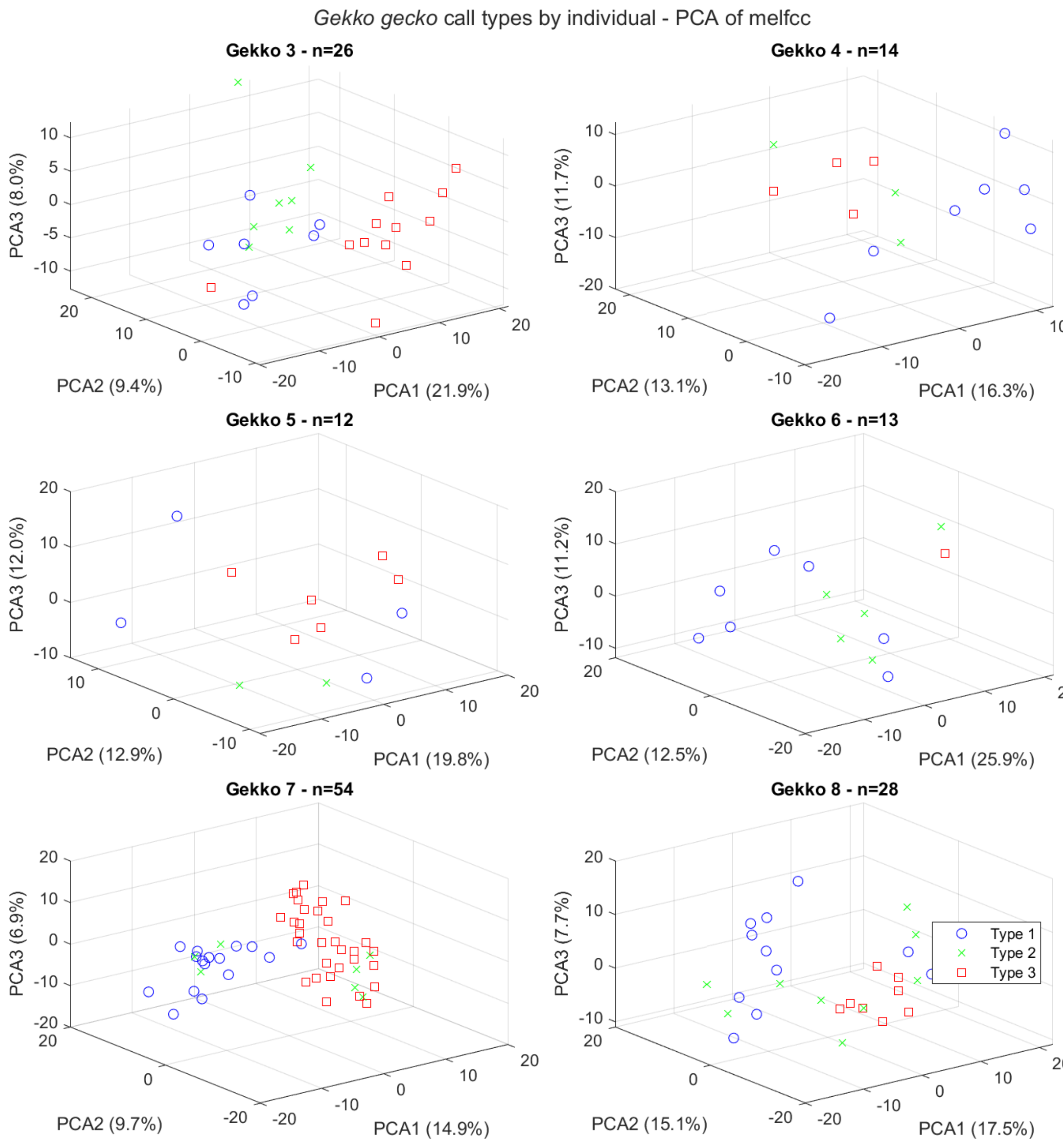

Fig. S2. Results of PCA on the MFCCs of vocalizations for each individual *Gekko gecko*.

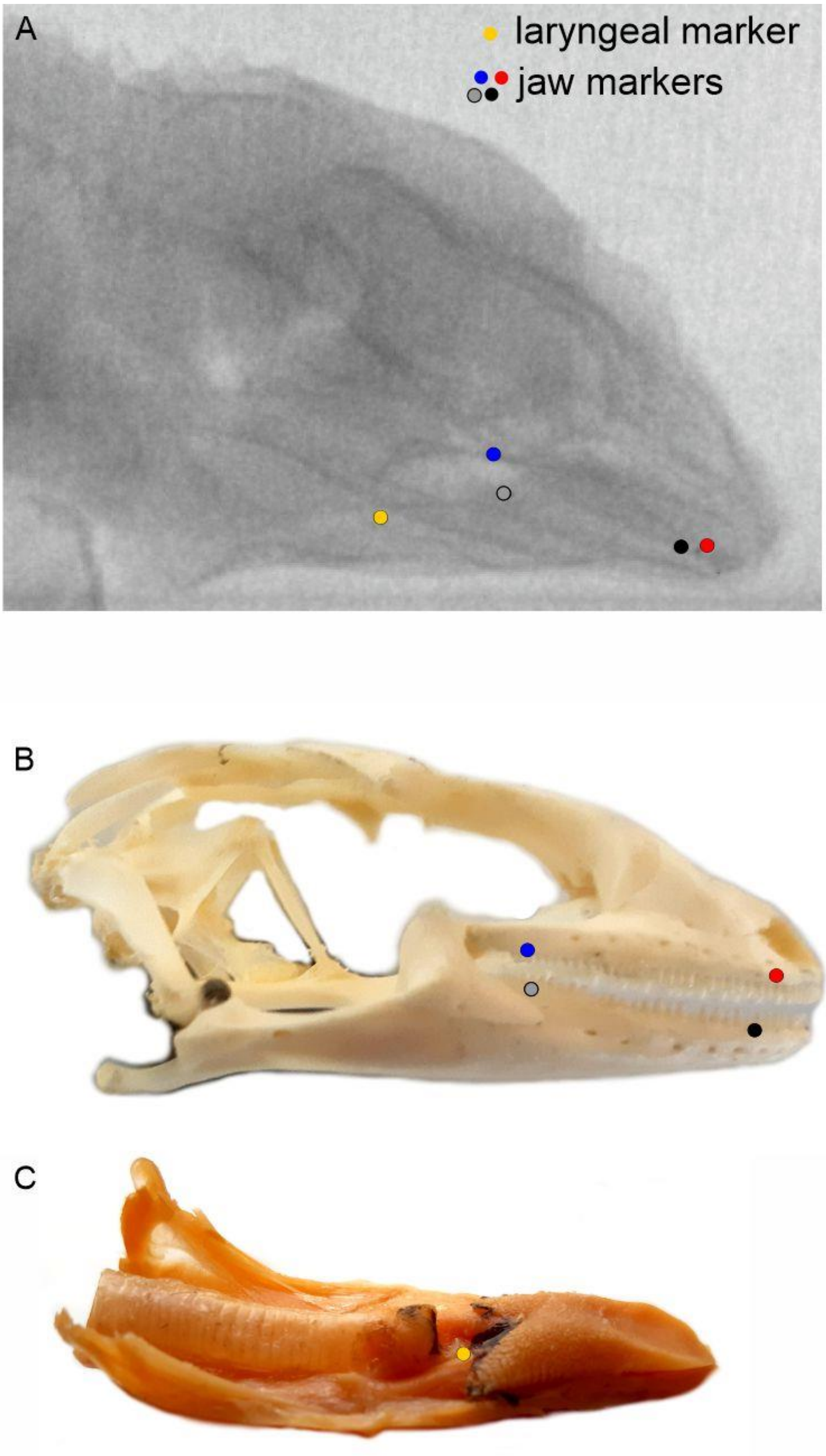

Fig. S3. (A) X-Ray image of a *Gekko gecko* individual, showing the implanted markers. (B) Schematic of a *Gekko gecko* skull as a reference with location of the implanted markers as an overlay. (C) Schematic of a *Gekko gecko* tongue with implanted laryngeal marker position as an overlay. Colors: blue, red, gray and black = skull markers, yellow = laryngeal marker.

Table S1. Distribution of number of call types produced by 6 Gekko gecko individuals.

Individual	Total	Type 1	Type 2	Type 3	Sex
**3**	32	9	7	16	male
**4**	14	7	3	4	male
**5**	12	4	2	6	male
**6**	14	7	5	2	male
**7**	56	16	8	32	male
**8**	28	11	9	8	female
**Sum**	**156**	**54**	**34**	**68**	

Table S2. Sample sizes used in different components of the study.

component of the study	number of animals used (N)	number of events observed (n)	number of events observed per individual	sex of animals
sound recordings	6	156	see supplementary table 1	5 males<br>1 female
PCA of sound recordings	6	147	see supplementary table 1	5 males<br>1 female
X-ray analysis (vocalization sequences)	2	10	5 sequences per animal	2 males
X-ray analysis (breathing sequences)	2	2	2 sequences per animal	2 males
absolute breathing events (lateral view)	2	11	animal 1 = 5 events<br>animal 2 = 6 events	2 males
absolute vocalization events (lateral view)	2	14	animal 1 = 14 events<br>animal 2 = 6 events	2 males
absolute breathing events (dorsal view)	2	13	animal 1 = 8 events<br>animal 2 = 5 events	2 males
absolute vocalization events (dorsal view)	2	25	animal 1 = 8 events<br>animal 2 = 17 events	2 males
neuronal backfills	4	4	1 backfill per animal	4 males

Journal of Experimental Biology • Supplementary information

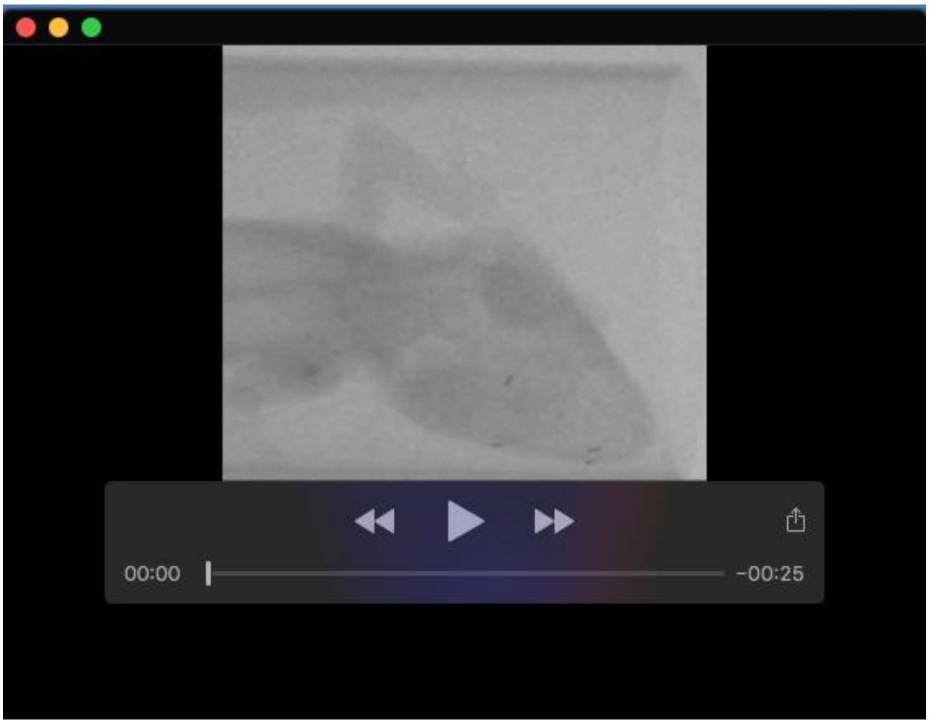

Movie 1. Displacement of jaw and laryngeal markers visualized by dorso-ventral and lateral cineradiography of a *Gekko gecko* during breathing.

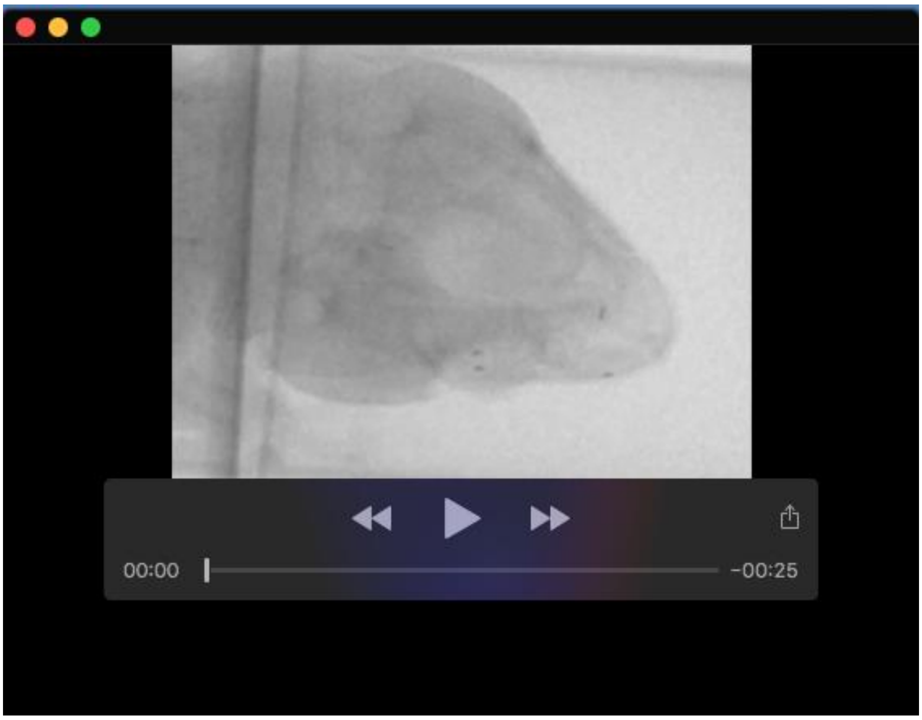

Movie 2. Displacement of jaw and laryngeal markers visualized by dorso-ventral and lateral cineradiography of a *Gekko gecko* during vocalization.
